# Obesity-Linked Gut Microbiome Dysbiosis Associated with Derangements in Gut Permeability and Intestinal Cellular Homeostasis Independent of Diet

**DOI:** 10.1155/2018/3462092

**Published:** 2018-09-03

**Authors:** Ravinder Nagpal, Tiffany M. Newman, Shaohua Wang, Shalini Jain, James F. Lovato, Hariom Yadav

**Affiliations:** ^1^Department of Internal Medicine-Molecular Medicine and Department of Microbiology and Immunology, Wake Forest School of Medicine, Winston-Salem, NC, USA; ^2^Division of Public Health Sciences, Wake Forest School of Medicine, Winston-Salem, NC, USA

## Abstract

This study aimed to determine the association between non-high-fat diet-induced obesity- (non-DIO-) associated gut microbiome dysbiosis with gut abnormalities like cellular turnover of intestinal cells, tight junctions, and mucin formation that can impact gut permeability. We used leptin-deficient (Lep^ob/ob^) mice in comparison to C57BL/6J control mice, which are fed on identical diets, and performed comparative and correlative analyses of gut microbiome composition, gut permeability, intestinal structural changes, tight junction-mucin formation, cellular turnover, and stemness genes. We found that obesity impacted cellular turnover of the intestine with increased cell death and cell survival/proliferation gene expression with enhanced stemness, which are associated with increased intestinal permeability, changes in villi/crypt length, and decreased expression of tight junctions and mucus synthesis genes along with dysbiotic gut microbiome signature. Obesity-induced gut microbiome dysbiosis is also associated with abnormal intestinal organoid formation characterized with decreased budding and higher stemness. Results suggest that non-DIO-associated gut microbiome dysbiosis is associated with changes in the intestinal cell death versus cell proliferation homeostasis and functions to control tight junctions and mucous synthesis-regulating gut permeability.

## 1. Introduction

Increased prevalence of obesity and its associated comorbidities around the world became epidemic, with constant rising rates indicating no successful prevention and therapeutic strategies available. This is because of complex pathophysiology of obesity that involves multifaceted interactions of genetics, diet, lifestyle, and environmental factors [1]. The contribution of gut microbiome perturbations (dysbiosis) in obesity pathology is now well known [2]; however, the mechanisms are not well defined. Diet is a major manipulator of gut microbiome; for example, high-fat diet- (HFD-) feeding develops microbiome dysbiosis in parallel to systemic abnormalities into the host metabolism causing obesity and type 2 diabetes [3]. It is evident that gut microbiome dysbiosis becomes apparent earlier than measurable host metabolic dysfunctions, suggesting that gut microbiome dysbiosis might be the primary factor involved in the progression of obesity and type 2 diabetes during high fat/calorie diets; however, contribution of non-high-fat diet-induced obesity (non-DIO) in intestinal dysfunctions and gut microbiome dysbiosis link is not well known. Leptin-deficient (Lep^ob/ob^) mice are a classical model of non-DIO that are developed while fed with normal chow but with overeating behavior. Gut microbiome dysbiosis also appears in Lep^ob/ob^ mice, similar to HFD-induced obesity, which is independent of dietary ingredient differences, suggesting that host obesity can also influence gut microbiome composition. Fecal microbiome transplantation from Lep^ob/ob^ and diet-induced obese (DIO) mice which causes obesity in recipients supports the notion that gut microbiome dysbiosis causes obesity [2]; however, mechanism(s) on how gut microbiome dysbiosis contributes in obesity progression are (is) still unknown. Proposed mechanisms like increased energy harvest [2] and increased inflammation [4] induced by gut microbiome dysbiosis are partially known and present limited information of how non-DIO is associated with gut dysfunctions. Gut permeability is increased in HFD-fed mice models that are characterized with increased inflammation linked to leaking microbial cell wall component lipopolysaccharide (LPS) of gram-negative bacteria causing endotoxemia [5]; however, non-DIO-induced gut leakiness and endotoxemia are not defined well. The intestine being a highly regenerating organ, where intestinal epithelia rejuvenate every 3–7 days, we hypothesize that obesity-induced gut microbiome dysbiosis may be associated with the abnormalities in cellular turnover (i.e., cell death versus survival/proliferation) that impact gut permeability via modulation of tight junction and mucin formation. Hence, the aim of present study was to define the association among non-DIO- (in Lep^ob/ob^ mice) linked gut microbiome dysbiosis with (a) cellular turnover homeostasis and structural maintenance of the intestine and (b) expression of tight junctions and mucus synthesis genes that regulates gut permeability.

## 2. Materials and Methods

### 2.1. Mice

About 8 weeks old leptin-deficient (Lep^ob/ob^) and C57BL/6J males (*n* = 12 in each group) were procured from Jackson Laboratory (Bar Harbor, ME, USA) and housed in a temperature-, humidity-, and light-controlled (12 hrs light-dark cycle) Wake Forest Animal Resource Program (ARP) facility for another 8 weeks. Both groups of mice were fed with identical diet (Prolab® RMH 3000 5P00^∗^ from Lab Diets Inc., St. Louis, MO), ad libitum. Fecal samples and body weight measures were collected weekly. After 16 weeks, the blood glucose, glucose, and insulin tolerance tests and area under the curve are calculated, as described in our earlier publications [6–8]. Thereafter, *n* = 3 animals from each group were euthanized using CO_2_ chamber, and the whole small intestine (from duodenum to ileum) has been used for organoid formations. The rest of the animals were used for measurement of gut permeability, intestinal histology, and gene expression analyses. All the animal experiments and procedures were approved by the IACUC of Wake Forest ARP.

### 2.2. Intestinal Organoid Formation and Culture

The entire small intestine of each mouse was cleaned by flushing with PBS and cut open lengthwise, washed, transferred to clean PBS and further cut into 2 mm segments. The intestinal segments were pipetted back and forth and allowed to settle. Washes were repeated ~20 times and segments were finally suspended in 25 mL Gentle Cell Dissociation Reagent (STEMCELL, #07174) for 15 min at room temperature on a rocking platform. After removing the Gentle Cell Dissociation Reagent, 10 mL of cold PBS with 0.1% BSA was added and the mixture was filtered through a 70 *μ*m filter, following 4 times wash. After centrifuging at 290 ×g for 5 min at 4°C, the pellets were resuspended in 10 mL of cold PBS with 0.1% BSA and centrifuged again at 200 ×g for 3 min. Pellets were resuspended in 10 mL cold DMEM/F12 and centrifuged at 200 ×g for 5 min followed by resuspending in 150 *μ*L IntestiCult Organoid Growth Medium (STEMCELL, #06005) with 50 *μ*g/mL gentamicin. The Matrigel Matrix (Corning, #354230) was added to the suspension, and the mixture (50 *μ*L) was plated in a prewarmed 24-well culture plate. The plate was placed in the incubator at 37°C and 5% CO_2_ for 10 min to allow the Matrigel to set, and 500 *μ*L of IntestiCult Organoid Growth Medium was added into each well. To maintain the cultures, the IntestiCult Organoid Growth Medium was changed three times/week, and organoids were viewed under the microscope on days 4, 6, 7, and 13. The organoids were separated into “nonbudding,” defined as having a singular, circular shape, and “budding,” defined as possessing lobe-like structures connected to the central body of the organoid. These groups were counted during each of the microscope observations. However, the number of buds per organoid was counted from only budding organoids to determine if the total number of bud formations is also changed in Lep^ob/ob^- versus control mice-derived organoids. On day 4, images were taken of approximately 20 organoids from each well. The images were assigned random numbers and were submitted for blind bud counting to another lab member.

### 2.3. Gut Permeability Assay

Half of the animals were used to measure gut permeability, while being deprived food access for 4 hrs, and then 4 kDa FITC-dextran (Sigma; 60 mg/100 g body weight) was given orally. After 4 hrs of ingestion, blood was collected from a tail vein and serum was separated by centrifuging the blood. Fluorescence intensity for FITC in the plasma was measured to determine the rate of gut leakiness of FITC-dextran [9].

### 2.4. Real-Time PCR

The expression of genes related to cell death (*Bad* and *Bax*), cell survival/proliferation (i.e. *Bcl2*, *Ccnd1*, *Cdk6*, and *Sox4*), intestinal stem cells (*Lgr5*, *Olfm4*, and *Bmi1*) [10], mucin biology (*Muc2* and *Muc6*), and tight junctions (*occludin*, *zonulin-1*, and *Jam*) was analyzed using real-time PCR (Table S1). Total RNA was isolated using RNeasy kit (Qiagen Inc., USA) and reverse transcribed using high-capacity reverse transcription kit (Applied Biosystems), further using cDNA for real-time PCR as described in our earlier studies. 18S rRNA was used as an internal control. Relative gene expression was calculated using ΔΔCT procedure and presented as relative fold change.

### 2.5. Western Blot Analysis

Intestinal tissue (ileum) was homogenized in homogenization buffer [6, 8], and Western blot analyses were performed to measure the tight junction proteins (e.g., Zo1 and occludin). Tubulin has been used as an internal loading control. Band intensities were calculated using ImageJ software and presented as fold change, while being normalized with tubulin content loaded in each well.

### 2.6. Histochemical Analysis

For histological analyses, intestinal tissues from mice were collected and washed with PBS followed by 10% formalin solution. Then intestinal tissues were dipped in 10% formalin overnight and were fixed on paraffin blocks and sections cut at 0.5 *μ*m thickness. Hematoxylin and eosin (H&E) staining was performed following standard methods, and pictures were taken using AmScope microscope on 20x magnification using 9MP digital camera. Villi length and intestinal wall thickness were measured using ImageJ software by a blinded person.

### 2.7. Gut Microbiome Analysis

Feces for microbiome analysis were immediately placed in sterile tubes under aseptic conditions, frozen, and stored at −80°C until further processing. DNA from samples was extracted by using the Qiagen DNA Stool Mini Kit (Qiagen, CA, USA), and the 16S rRNA gene was amplified using primers 515F (barcoded) and 806R, which flanked the V4 hypervariable region of bacterial 16S rRNAs, following the Earth Microbiome Project protocol [11–13]. After PCR reaction, amplicons were purified using Agencourt® AMPure® XP (Beckman Coulter), quantified using the Qubit 3 fluorimeter (Invitrogen), normalized to an equal concentration (4 nm) and pooled together for sequencing on an Illumina MiSeq platform [12].

The 16S rRNA gene amplicons were demultiplexed, quality-filtered, and clustered using default parameters in Quantitative Insights Into Microbial Ecology (QIIME version 1.8.0), software that enables microbial community analysis [11]. Sequences were grouped into operational taxonomic units (OTUs) at a sequence similarity of 97% and assigned to OTUs through the open-reference picking software UCLUST in QIIME. Taxonomy assignment and diversity analyses including observed OTUs and Shannon, Chao1, and PD Whole Tree diversity indices were computed through QIIME with default settings to compare bacterial species richness between the two groups. Bacterial composition of each sample was measured at various taxonomic levels (phylum through genus) using QIIME. Alpha and beta diversities were generated within QIIME by using weighted and unweighted UniFrac distance matrices. UniFrac distances are appraised as the distance between bacterial communities explaining the phylogenetic relationship between bacteria [14].

### 2.8. Statistical Analysis

All the values presented here are mean ± standard error of means. Student *t*-test and post hoc ANOVA analyses were performed to find statistical significance. *p* values with <0.05 are defined as significantly different. The data of bacterial diversity and abundance between the two groups were compared by using nonparametric analyses in the R statistical software package (version 3.4.2; https://www.r-project.org/), wherein significant differences were calculated by Kruskal–Wallis or Mann–Whitney test followed by Monte Carlo permutation. Unless otherwise stated, a value of *p* < 0.05 was considered statistically significant. Nonparametric Spearman correlation coefficients between biome and mice characteristics are presented with *p* values against the null hypothesis of zero correlation.

## 3. Results

### 3.1. Increased Gut Permeability in Lep^ob/ob^ Mice Associated with Reduced Tight Junction and Mucin Gene Expression

**Figure 1:**
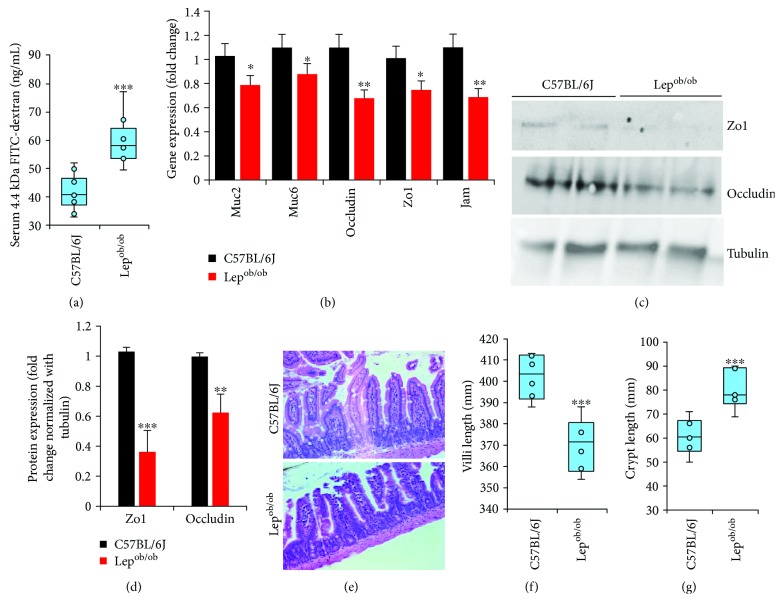
Gut permeability and intestinal structural changes are associated with mucin-tight junction formation independent of dietary differences. (a) Gut permeability (diffusion of FITC-dextran; 4 KDa from gut to blood) increased obese mice compared to B6 control. (b) Expression of mucin (Muc2 and Muc6) and tight junction- (occludin-, Zo1-, and Jam-) forming genes significantly decreased in Lep^ob/ob^ intestine than B6 mice. (c, d) Western blot analyses of Zo1, occludin, and tubulin in intestinal tissues. (e–g) Hematoxylin and eosin (20x) staining for the small intestine (ileum) (e), decreased length of villi (f), and increased crypt (g) in obese mice. Values presented here are average ± SEM. *p* values are defined as ^∗^ < 0.05, ^∗∗^ < 0.01, and ^∗∗∗^ < 0.001.

Significant increase in gut permeability (indicated by increased diffusion of 4KDa FITC-dextran into the blood from the gut) was observed in Lep^ob/ob^ mice compared to B6 control (Figure 1(a)). The expression of tight junction proteins like (mRNA and protein) occludin, zonulin-1 (Zo1), and junctional adhesion molecule (Jam), as well as mucin synthesis gene mRNAs (Muc2 and Muc6), was significantly decreased in Lep^ob/ob^ mice (Figures 1(b)–1(d)), suggesting that obesity resulting from leptin deficiency decreased expression of tight junction and mucin synthesis genes, which might result to leaky gut with increased permeability.

### 3.2. Obesity Is Associated with Structural Derangements in Intestine and Intestinal Organoids

**Figure 2:**
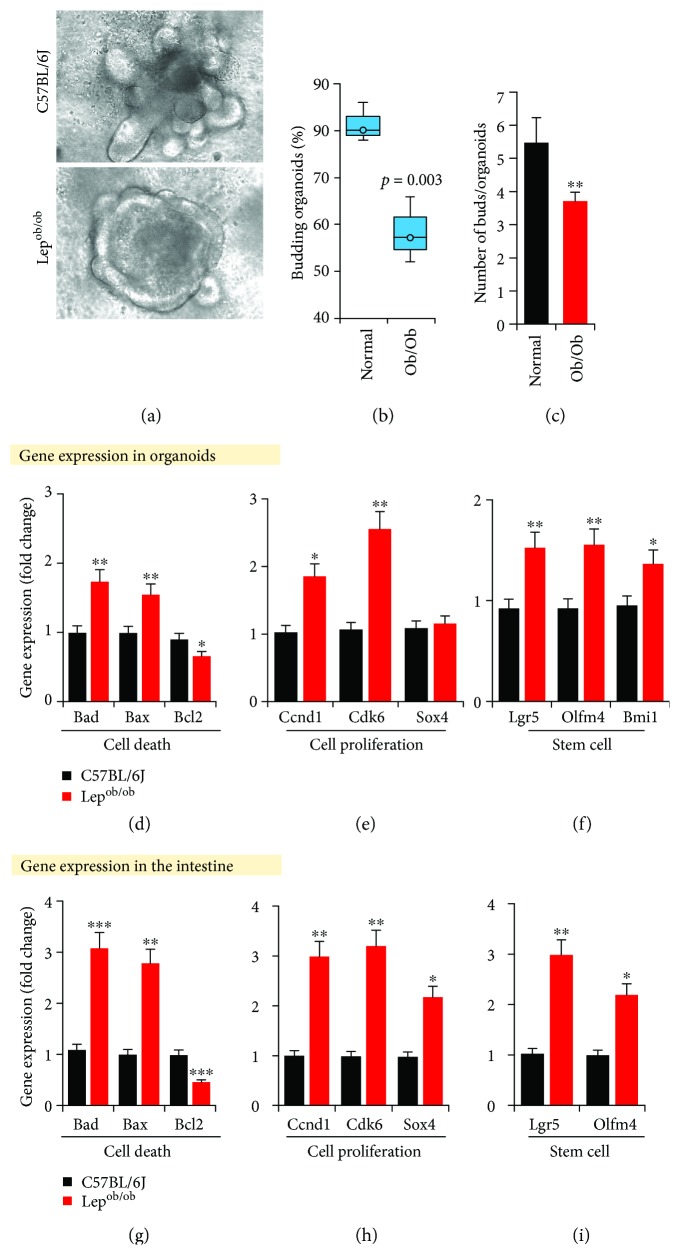
Non-diet-induced obesity developed stable changes in cells to form abnormal intestinal structures and influenced intestinal cellular homeostasis. (a–c) Intestinal organoids (a) and their budding potential (b, c) decreased in Lep^ob/ob^-derived organoids versus B6 mice. (d–i) mRNA expression of cell death (*Bad*, *Bax*, and *Bcl2*), cell proliferation (Ccnd1, Cdk6, and Sox4), and stem cell- (Lgr5-, Olfm4-, and Bmi1-) specific genes in obese and B6 intestines. Values presented here are average ± SEM/SD. *p* values are defined as ^∗^ < 0.05, ^∗∗^ < 0.01, and ^∗∗∗^ < 0.001.

Villi length was significantly decreased, while crypt length was increased in Lep^ob/ob^ mice (Figures 1(e)–1(g)), suggesting obesity induced significant structural derangements into the intestinal tissues, without difference in dietary ingredients that mice are eating. To further investigate the impact of obesity on the capacity of intestinal cells to form and maintain intestinal structural architecture, we analyzed the intestinal organoids prepared from Lep^ob/ob^ and normal mice and found that the budding capacity and number of total buds (in budding organoids) significantly decreased in Lep^ob/ob^-derived organoids (Figures 2(a)–2(c)). Central volumetric area was increased in obese-derived organoids compared to normal (data not shown here), suggesting that obesity was associated in creating stable changes in the intestinal cells that contribute in abnormal organization of the intestinal structure.

### 3.3. Cellular Turnover and Stemness Increased in Lep^ob/ob^ Intestine

To further determine the impact of obesity on intestinal cellular homeostasis that maintains tissue structure and integrity, we measured the expression of cell death/survival, proliferation, and stem cell genes in the organoids and intestinal tissue. We found that the expression of both the cell death (*Bad* and *Bax*) and cell proliferation (*Ccnd1*, *Cdk6*, and *Sox4*) genes was significantly increased in the organoids and intestine of Lep^ob/ob^ mice compared to normal (Figures 2(d), 2(e), 2(g), and 2(h)). However, expression of the cell survival gene (*Bcl2*) was significantly decreased in obese (Figures 2(d) and 2(g)). In addition, the stem cell markers like leucine-rich repeat-containing G-protein coupled receptor 5 (*Lgr5*), olfactomedin 4 (*Olfm4*), and BMI1 (protooncogene, polycomb ring finger) expression increased in the Lep^ob/ob^ intestine as well as their organoids (Figures 2(f) and 2(i)), suggesting that obesity-associated gut microbiome dysbiosis exposure is associated with faster/abnormal cellular turnover of intestinal cells, along with increased stemness [15].

### 3.4. Gut Microbiome Dysbiosis and Metabolic Abnormalities Are Associated in Lep^ob/ob^ Mice That Are Independent of Diet Differences

**Figure 3:**
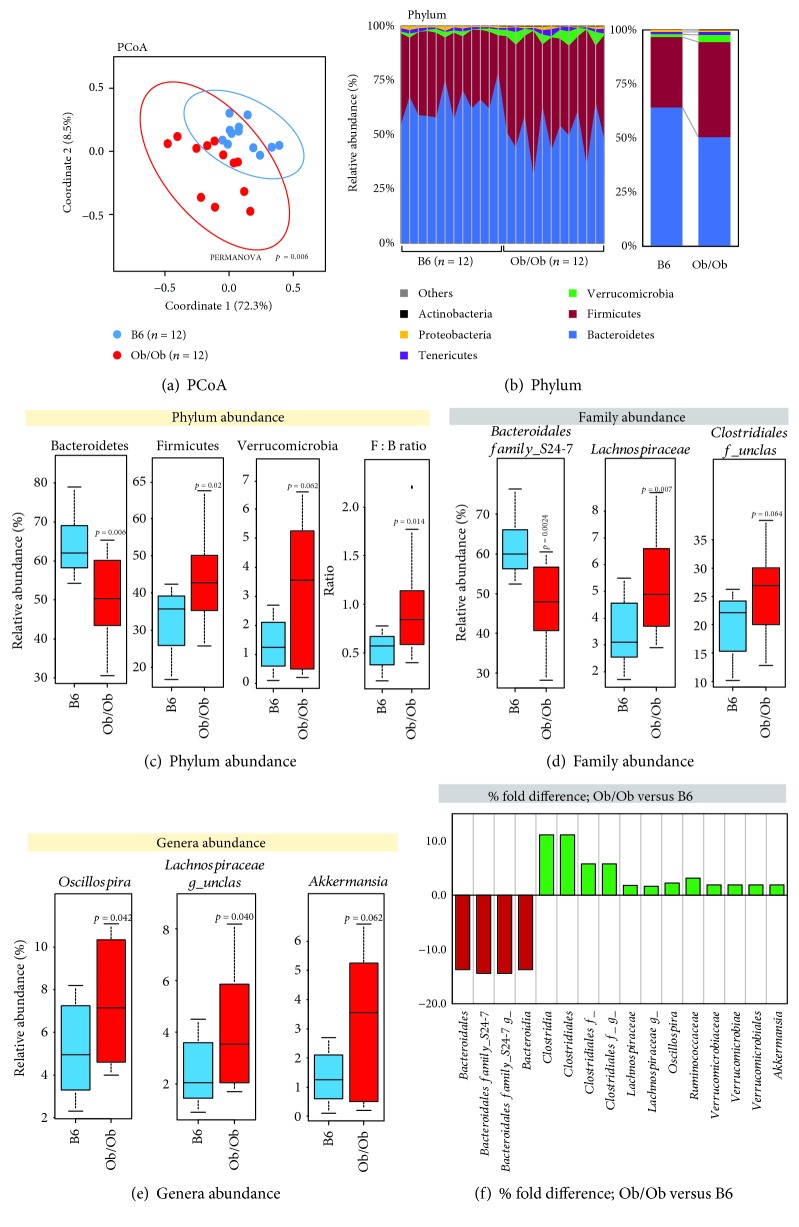
Lep^ob/ob^ mice exhibit significant gut microbiome dysbiosis compared to C57BL/6J (B6) mice fed with identical diet. (a) PCoA analysis shows differential clustering of the microbiome signature in Lep^ob/ob^ and B6 mice. (b, c) Microbial phylum abundance and Firmicutes : Bacteroidetes (F : B) ratio in Lep^ob/ob^ and B6 mice. (d–f) Significantly differing microbial families (d) and genera (e) abundances as well as fold change (percent) between Lep^ob/ob^ and B6 mice. Values presented here are average ± SEM/SD.

Lep^ob/ob^ mice were more than 2 times heavier than control mice (54.2 ± 5.7 gm versus 26.4 ± 1.8 gm) at the age of 16 weeks, along with significantly higher fasting and fed blood glucose levels (Supplementary Figures 1a and b). During GTT and ITT, glucose clearance rate was significantly lower (Supplementary Figures 1d and e) suggesting abnormal glucose homeostasis and increased insulin resistance. Microbiome analysis using PCoA alignment showed a differentially skewed microbiome signature between Lep^ob/ob^ and normal mice (Figure 3(a)). Although we have not seen differences in phylogenetic diversity (PD), Chao1, and observed species indices, the Shannon index (a measure of microbial diversity) was increased in Lep^ob/ob^ mice compared to normal mice (Supplementary Figure S2). Taxonomic analysis of phylum distribution shows significant increase in the Firmicutes population and decreased Bacteroidetes in Lep^ob/ob^ mice compared to control, which is also shown by the increased Firmicutes : Bacteroidetes (F : B) ratio between the two groups (Figures 3(b) and 3(c)). *Verrucomicrobia* was found increased in Lep^ob/ob^ mice compared to normal but did not achieve statistically significant differences. In-depth family-level taxonomic analyses show that *Bacteroidales family_S24-7* decreased in Lep^ob/ob^ while *Lachnospiraceae* and *Clostridiales family_unclassified* increased compared to control mice (Figures 3(d) and 3(e)). In addition, *Oscillospira* and *Lachnospiraceae genus_unclassified* increased in Lep^ob/ob^ versus control mice, with an increased trend in *Akkermansia* abundance but not reaching statistically significant differences (Figures 3(e) and 3(f); Table S2). These results suggest that obesity itself has profound effects on gut microbiome, independent of diet, that may contribute in the pathogenesis of obesity and its related illnesses like insulin resistance.

### 3.5. Obese Gut Microbiome Dysbiosis Is Associated with Intestinal Cellular Homeostasis Regulating Gut Permeability and Mucin Biology

**Figure 4:**
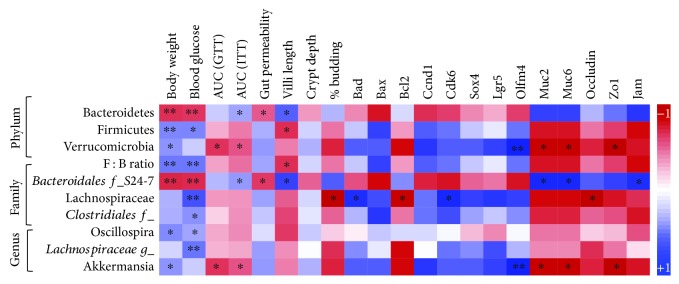
Relationship of metabolic functions, intestinal morphology, and functions with gut microbiome dysbiosis, independent of dietary differences. Spearman correlation analysis to establish the association between metabolic measures (body weight, blood glucose, AUC-GTT, and AUC-ITT), gut permeability changes, intestinal structural changes, and gene expression changes and the gut microbiome signature between Lep^ob/ob^ and B6 mice. Values presented here are average of 3–8 replicates. *p* values are defined as ^∗^ < 0.05 and ^∗∗^ < 0.01.

Our correlation analyses (Figure 4, Table S3) suggest that the abundance of Bacteroidetes are negatively, while abundance of Firmicutes and Verrucomicrobia are positively, correlated with body weight, fasting blood glucose, area under the curve (AUC) during glucose tolerance (GTT), gut permeability, crypt length, percent of nonbudding organoids, cell death (*Bad* and *Bax*), proliferation (*Ccnd1*, *Cdk6*, and *Sox4*), and stem cells (*Lgr5* and *Olfm4*), suggesting that decreased Bacteroidetes abundance and increased Firmicutes and Verrucomicrobia abundance are associated with the detrimental status of metabolic measures and intestinal cellular homeostasis, whereas AUC during insulin tolerance test (indicator of insulin resistance), villi length, percent of organoid budding, cell survival (*Bcl2*), mucin synthesis genes (*Muc2* and *Muc6*), and tight junction genes (*occludin*, *Zo1*, and *Jam*) are positively associated with Bacteroidetes but negatively correlated with Firmicutes and Verrucomicrobia, suggesting a detrimental link of Firmicutes and Verrucomicrobia with intestinal measures. The family of *Bacteroidiales family_S24-7* abundance has similar correlation with the Bacteroidetes phylum, while the family of *Lachnospiraceae* and *Clostridiales family_unclassified* abundance and genus of *Oscillospira* and *Lachnospiraceae genus_unclassified* and *Akkermansia g-unclassified* abundance followed similar correlation with Firmicutes and Verrucomicrobia with metabolic, gut permeability, intestinal structural, and cell homeostasis genetic markers, further suggesting the close link between gut microbiome dysbiosis, and intestinal structural and functional changes are associated with obesity that are independent of dietary differences.

## 4. Discussion

High calorie/fat diets remain one of the major contributions for creating gut microbiome dysbiosis and obesity and derangements into the intestinal tissues [16]; however, the impact of diet-independent obesity on gut microbiome dysbiosis and abnormal structural and functional changes into the intestinal tissues are not well known. High cell turnover (cell death and proliferation) occurring in the intestine seed the possibility that the gut microbiome and its metabolites may impact the cellular homeostasis of intestinal epithelial cells. Here, we established the association between non-high-fat diet-induced obesity- (non-DIO-) caused gut microbiome dysbiosis and disturbances in the cellular homeostasis of intestinal tissues to preserve gut integrity, independent of dietary differences. As found by others [2], we also observed significant changes in the gut microbiome signature in Lep^ob/ob^ mice compared to conventional normal mice. Interestingly, obese mice showed significant increased abundance of *Lachnospiraceae* bacteria that has been known to enhance non-diet-induced insulin resistance/type 2 diabetes in obese mice [17]. Although *Akkermansia* abundance influenced significantly in Metformin- (antidiabetic medicine-) treated patients as well as being known to be reduced in HFD-induced obesity [18, 19], our studies show that abundance of this group of bacteria is increased in Lep^ob/ob^ mice, suggesting that depletion of *Akkermansia* in obese humans might be associated with high fat diet intake.

Increased gut permeability is a common cause of HFD-induced obesity that results in increasing the leakiness of bacterial substances like LPS that can diffuse through the mucous layer and tight junctions [5, 20] and can interact with host immune cells to cause low-grade inflammation, which is the characteristics of obesity and insulin resistance [21]. Our studies show that obesity is associated with increased gut permeability independent of dietary differences, suggesting that the obesity might primarily influence gut permeability that may result in increased inflammation or endotoxemia without consuming HFD. In addition, our results also suggest that non-DIO and gut microbiome dysbiosis also associated with decreased expression of tight junction proteins (*occludin* and *Zo1*) and mucin synthesis genes (*Muc2* and *Muc6*) further support that obesity and the associated gut microbiome dysbiosis contribute in pathology of gut leakiness. However, these results are unable to explain how non-DIO-associated gut microbiome dysbiosis causes abnormalities in a tight junction and mucin synthesis gene expression, which remains to be elucidated in further studies. In addition, leptin is an important adipokine secreted from adipose tissue and known for regulating a myriad of cellular functions, that is, apoptosis and antimicrobial peptide secretion in the gut [22, 23]. Here, we used leptin-deficient mice (Lep^ob/ob^); hence, it may be plausible that leptin deficiency may contribute in the changes we observed in cellular turnover into the gut. Such a hypothesis is also supported by evidences showing that leptin levels are positively correlated with insulin-like growth factor-1 (IGF1) and IGF1-binding protein 3 (IGFBP3) levels in other systems [24, 25] and recent studies by D'Addio et al. [26] showing that IGF1/IGFBP3 controls intestinal stem cell growth and differentiation. This suggests that the leptin-IGF1/IGFBP3 axis may play an important role in development of intestinal cellular biology. However, further comprehensive studies will be needed to establish the contribution of the obesity-associated leptin-IGF1/IGBP3 axis in intestinal stem cell biology and decipher the causal or correlational link among these pathways.

Intestinal epithelia interspersed with diverse types of cells regenerate every 3–7 days, and these cells attach with tight junction proteins and form a physical barrier to prevent leaking of intestinal contents. In addition, goblet cells secrete mucous to overlay the intestinal epithelium that further makes a barrier to filter the leakiness and direct interaction of gut microbiome and its ingredients to the host cells [21]. However, the link of obesity-associated gut microbiome dysbiosis on cell death and proliferation is not known. Our results showed an important clue that obesity-associated gut microbiome dysbiosis associated with enhanced/abnormal cellular turnover into the intestinal tissue. Increased cell death that is compensated with increased cell proliferation seen in our studies might have impacted the formation of tight junctions and mucin-producing cells that may have resulted in increased gut permeability. Furthermore, changes in cell death and proliferation balance in intestinal epithelia may yield structural changes in intestinal tissues. For example, we found that stem cell markers and crypt length in the Lep^ob/ob^ intestine is increased, while budding capacity and number of buds in Lep^ob/ob^-derived organoids significantly decreased, suggesting that obesity created stable changes in stem cell pools of crypt that are unable to make normal intestinal structures, especially in in vitro conditions. However, Lep^ob/ob^-derived organoids form a bigger central lumen, further suggesting that the obese-derived organoids proliferate to make a more straight epithelial wall rather than making organized structures like crypt/villi that may have functional consequences for intestinal tissues. In addition, increased stemness markers in the obese intestine further indicate the association of obesity and intestinal cancers that are associated with high-fat diet-induced obesity [15] and gut microbiome dysbiosis [2].

However, here, we still do not know who contributed primarily in such changes, obesity or gut microbiome dysbiosis, and such causal versus consequential effects warrant further study. However, such effects are clearly independent of dietary ingredient differences. Therefore, there is much that we must find out to clarify the association between obesity, the microbiota, and gut permeability that can contribute in abnormal gut function and cellular homeostasis.

## 5. Conclusions

Contribution of gut microbiome in obesity pathophysiology is known; however, its mechanism(s) of action is (are) not well defined. Our study established a close link between obesity-associated gut microbiome dysbiosis to cause derangements in the intestinal cellular turnover homeostasis and functions to regulate gut permeability, independent of dietary ingredients like high fats. Although our studies still have limitations to explain causative versus consequential relationship for changes in cellular turnover induced by microbiome dysbiosis or vice versa, our results strongly support the basis to investigate the role of nutrient-microbiome-gene interactions to modulate gut physiology in pathology of obesity.

## Data Availability

All the data created and used to support the findings of this study are included within the article and supplementary information file(s). However, any additional data, to support findings of this study, are available from the corresponding author upon request.

## Supplementary Materials

Supplementary Materials Supplementary Figure S1: body weight (a), fasting (b), and fed (c) blood glucose levels of Lep^ob/ob^ and C57BL/6J mice (*n* = 8 in each group). Glucose tolerance test (d) and insulin tolerance test (e), as well as area under the curve during GTT (f) and ITT (g), of Lep^ob/ob^ and B6 mice. Values presented here are average ± SEM/SD. *p* values are defined as ^∗^ < 0.05, ^∗∗^ < 0.01, and ^∗∗∗^ < 0.001. Supplementary Figure S2: alpha diversity indices like phylogenetic degree (PD) tree (a), Chao1 (b), observed OTUs (c), and Shannon index (d) of gut microbiome from Lep^ob/ob^ and C57BL/6J mice. Table S1: primer sequences (mouse) used for real-time PCR (adopted from: PrimerBank: https://pga.mgh.harvard.edu/primerbank/). Table S2: relative abundance of different microbial communities that are significantly different in Lep^ob/ob^ versus C57BL/6J (B6) mice. Table S3: Spearman correlation analysis among metabolic, gut permeability, intestinal structural, and gene expression analyses with gut microbiome signature of Lep^ob/ob^ and C57BL/6J mice.
