# Supplementary material for: Obesity-Linked Gut Microbiome Dysbiosis Associated with Derangements in Gut Permeability and Intestinal Cellular Homeostasis Independent of Diet
Source: J Diabetes Res. 2018 Sep 3;2018:3462092. doi: 10.1155/2018/3462092 (PMC6140100; doi:10.1155/2018/3462092)
Supplement: Supplementary Materials — Supplementary Figure S1: body weight (a), fasting (b), and fed (c) blood glucose levels of Lepob/ob and C57BL/6J mice (n = 8 in each group). Glucose tolerance test (d) and insulin tolerance test (e), as well as area under the curve during GTT (f) and ITT (g), of Lepob/ob and B6 mice. Values presented here are average ± SEM/SD. p values are defined as ∗ < 0.05, ∗∗ < 0.01, and ∗∗∗ < 0.001. Supplementary Figure S2: alpha diversity indices like phylogenetic degree (PD) tree (a), Chao1 (b), observed OTUs (c), and Shannon index (d) of gut microbiome from Lepob/ob and C57BL/6J mice. Table S1: primer sequences (mouse) used for real-time PCR (adopted from: PrimerBank: https://pga.mgh.harvard.edu/primerbank/). Table S2: relative abundance of different microbial communities that are significantly different in Lepob/ob versus C57BL/6J (B6) mice. Table S3: Spearman correlation analysis among metabolic, gut permeability, intestinal structural, and gene expression analyses with gut microbiome signature of Lepob/ob and C57BL/6J mice. [file 3462092.f1.pdf]

Supplementary Figure S1

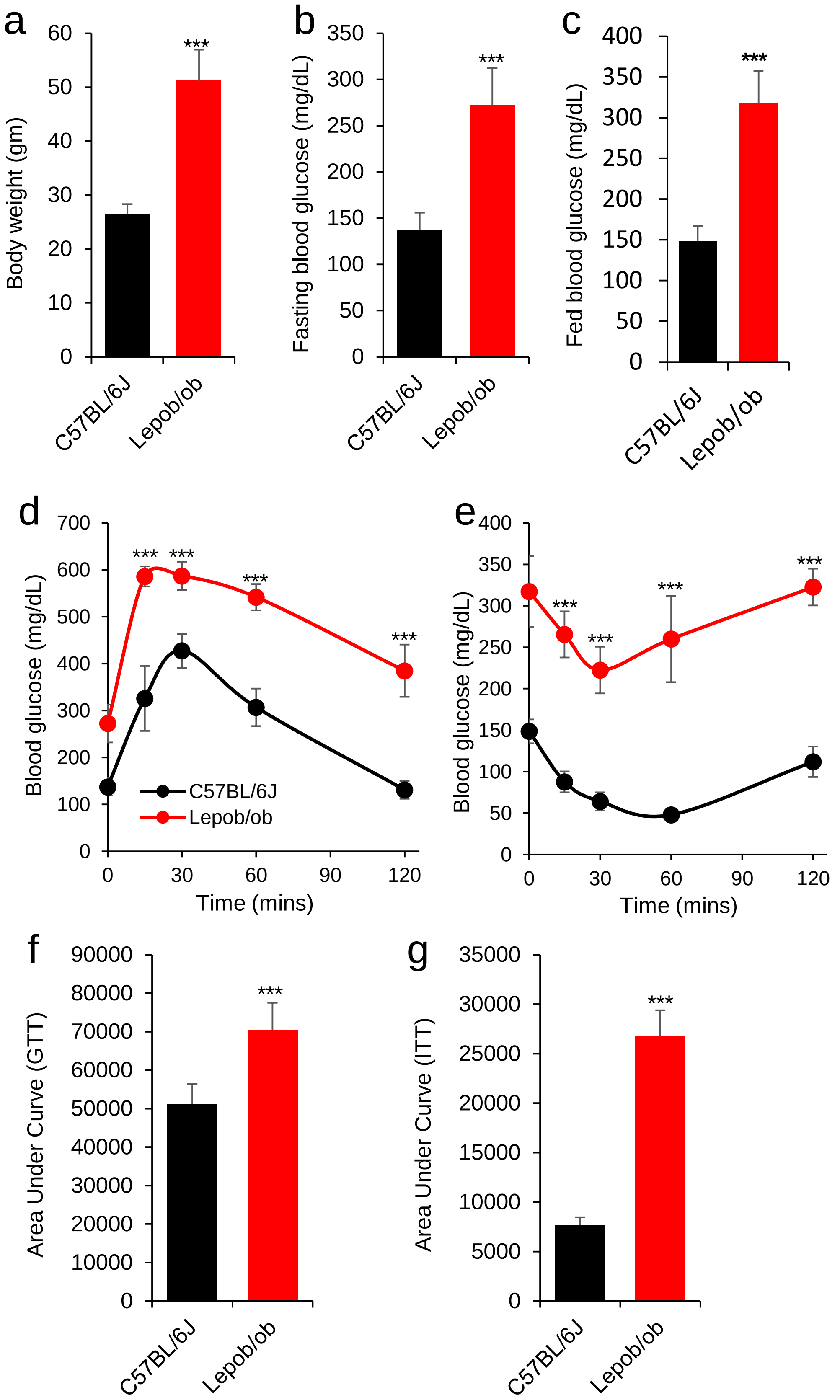

Supplementary Figure S2

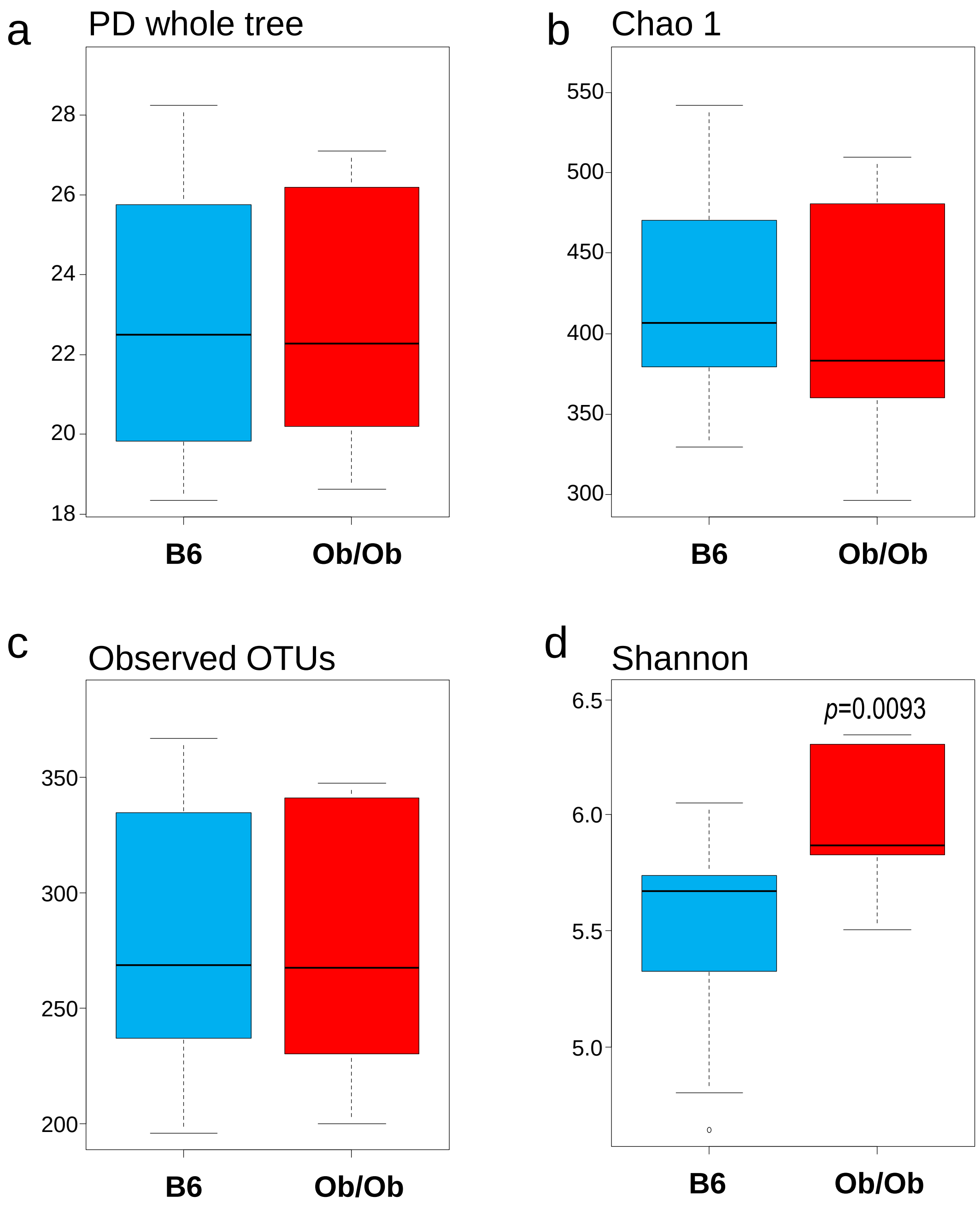

Mann-Whitney U test

Table S1: Primer sequences (mouse) used for real-time PCR (adopted from: PrimerBank: https://pga.mgh.harvard.edu/primerbank/)

**Gene name**	**Forward primer (5'→3')**	**Reverse primer (5'→3')**
Bax	TGAAGACAGGGGCCTTTTTG	AATTCGCCGGAGACACTCG
Bad	AAGTCCGATCCCGGAATCC	GCTCACTCGGCTCAAACTCT
Bcl2	ATGCCTTTGTGGAACTATATGGC	GGTATGCACCCAGAGTGATGC
Ccnd1	GCGTACCCTGACACCAATCTC	CTCCTCTTCGCACTTCTGCT
Cdk6	GGCGTACCCACAGAAACCATA	AGGTAAGGGCCATCTGAAAACT
Sox4	CGGCTGCATCGTTCTCTCC	GGTAGACGTGCTTCACTTTCTTG
Lgr5	CCTACTCGAAGACTTACCCAGT	GCATTGGGGTGAATGATAGCA
Olfm4	CAGCCACTTTCCAATTTCACTG	GCTGGACATACTCCTTCACCTTA
Bmi1	ATCCCCACTTAATGTGTGTCCT	CTTGCTGGTCTCCAAGTAACG
Muc2	ATGCCCACCTCCTCAAAGAC	GTAGTTTCCGTTGGAACAGTGAA
Muc6	CGGCTGCGTCTGTCCTAAG	GCATAGTCACATGGGCATTCC
Occludin (Ocln)	TTGAAAGTCCACCTCCTTACAGA	CCGGATAAAAAGAGTACGCTGG
Zo1 (Tjp1)	CCGCTAAGAGCACAGCAA	TCCCCACTCTGAAAATGAGGA
Jam	TCTCTTCACGTCTATGATCCTGG	TTTGATGGACTCGTTCTCGGG
18S rRNA	GTAACCCGTTGAACCCCATT	CCATCCAATCGGTAGTAGCG

Table S2: Relative abundance of different microbial communities that significantly different in Lepob/ob versus C57BL/6J (B6) mice

	**B6**		**Ob/Ob**		
	**Avg**	**SD**	**Avg**	**SD**	**P value (T-test)**
	**Relative abundance, %**				
Bacteroidales	64.16	7.76	50.43	10.94	0.0018
Bacteroidales;f__S24-7	61.81	7.50	47.38	10.35	0.0008
Bacteroidales;f__S24-7;g__	61.81	7.50	47.38	10.35	0.0008
Bacteroidia	64.16	7.76	50.43	10.94	0.0018
Clostridia	31.92	8.74	43.01	13.11	0.0233
Clostridiales	31.92	8.74	43.01	13.11	0.0233
Clostridiales;f__	20.12	5.54	25.88	7.87	0.0502
Clostridiales;f__;g__	20.12	5.54	25.88	7.87	0.0502
Lachnospiraceae	3.38	1.26	5.18	1.83	0.0102
Lachnospiraceae;g__	2.40	1.23	4.04	2.22	0.0354
Oscillospira	5.17	2.11	7.38	2.83	0.0412
Ruminococcaceae	7.98	3.00	11.10	4.42	0.0550
Verrucomicrobiaceae	1.33	0.92	3.23	2.42	0.0227
Verrucomicrobiae	1.33	0.92	3.23	2.42	0.0227
Verrucomicrobiales	1.33	0.92	3.23	2.42	0.0227
Akkermansia	1.33	0.92	3.23	2.42	0.0227
Pedobacter kwangyangensis	2.10	1.55	7.49	2.51	0.0000
Blautia hansenii	2.65	1.24	5.19	1.73	0.0004
Akkermansia muciniphila	1.17	0.76	3.19	2.43	0.0163
Alkaliphilus crotonatoxidans	2.78	1.08	0.80	0.35	0.0000

Table S3: Spearman correlation analysis among metabolic, gut permeability, intestinal structural and gene expression analysis with gut microbiome signature of Lepob/ob and C57BL/6J mice.

Prob > |r| under H0: Rho=0
Number of Observations

		Body_ weight	Blood_ glucose	AUC__GTT_	AUC__ITT_	Gut_permeability	Villi_ length	Crypt_ depth	__budding	Bad	Bax
Bacteroidetes	r	-0.59504	-0.57432	0.29963	0.41844	-0.45722	0.58844	-0.25919	0.37143	-0.31429	-0.71429
Bacteroidetes	p	0.0022	0.0033	0.1549	0.0419	0.0491	0.0441	0.4159	0.4685	0.5441	0.1108
	n	24	24	24	24	19	12	12	6	6	6
Firmicutes	r	0.50054	0.50435	-0.18870	-0.35225	0.39123	-0.60245	0.28021	-0.37143	0.31429	0.71429
Firmicutes	p	0.0127	0.0120	0.3772	0.0914	0.0977	0.0382	0.3777	0.4685	0.5441	0.1108
	n	24	24	24	24	19	12	12	6	6	6
Verrucomicrobia	r	0.46899	0.29508	-0.58268	-0.47138	0.45391	-0.34676	0.38529	-0.65714	0.60000	0.60000
Verrucomicrobia	p	0.0208	0.1616	0.0028	0.0201	0.0509	0.2695	0.2161	0.1562	0.2080	0.2080
	n	24	24	24	24	19	12	12	6	6	6
F_B_ratio	r	0.53165	0.53700	-0.23097	-0.38242	0.41860	-0.59649	0.27018	-0.37143	0.31429	0.71429
F-B ratio	p	0.0075	0.0068	0.2775	0.0651	0.0745	0.0406	0.3957	0.4685	0.5441	0.1108
	n	24	24	24	24	19	12	12	6	6	6
Bacteroidales_f__S24_7	r	-0.63188	-0.61445	0.36522	0.45532	-0.56316	0.70753	-0.42732	0.60000	-0.48571	-0.77143
Bacteroidales;f__S24-7	p	0.0009	0.0014	0.0793	0.0254	0.0121	0.0101	0.1659	0.2080	0.3287	0.0724
	n	24	24	24	24	19	12	12	6	6	6
Lachnospiraceae	r	0.38555	0.64032	-0.25973	-0.27589	0.43108	-0.44483	0.26270	-0.94286	0.82857	0.65714
Lachnospiraceae	p	0.0628	0.0008	0.2203	0.1919	0.0654	0.1473	0.4094	0.0048	0.0416	0.1562
	n	24	24	24	24	19	12	12	6	6	6
Clostridiales_f__	r	0.39182	0.47259	-0.07217	-0.28484	0.31754	-0.52189	0.21366	-0.42857	0.37143	0.77143
Clostridiales;f__	p	0.0583	0.0197	0.7375	0.1773	0.1852	0.0818	0.5049	0.3965	0.4685	0.0724
	n	24	24	24	24	19	12	12	6	6	6
Oscillospira	r	0.49043	0.41694	-0.20448	-0.30527	0.24232	-0.56140	0.30175	-0.08571	0.02857	0.31429
Oscillospira	p	0.0150	0.0427	0.3378	0.1469	0.3175	0.0575	0.3405	0.8717	0.9572	0.5441
	n	24	24	24	24	19	12	12	6	6	6
Lachnospiraceae_g__	r	0.27099	0.53928	-0.17221	-0.21183	0.44142	-0.29072	0.12259	-0.60000	0.48571	0.20000
Lachnospiraceae;g__	p	0.2003	0.0065	0.4210	0.3204	0.0585	0.3593	0.7043	0.2080	0.3287	0.7040
	n	24	24	24	24	19	12	12	6	6	6
Akkermansia	r	0.46899	0.29508	-0.58268	-0.47138	0.45391	-0.34676	0.38529	-0.65714	0.60000	0.60000
Akkermansia	p	0.0208	0.1616	0.0028	0.0201	0.0509	0.2695	0.2161	0.1562	0.2080	0.2080
	n	24	24	24	24	19	12	12	6	6	6

Spearman Correlation Coefficients
Prob > |r| under H0: Rho=0
Number of Observations

		Bcl2	Ccnd1	Cdk6	Sox4	Lgr5	Olfm4	Muc2	Muc6	Occludin	Zo1	Jam
Bacteroidetes	r	0.25714	-0.60000	-0.54286	-0.31429	-0.20000	-0.54286	0.71429	0.71429	0.37143	0.54286	0.77143
Bacteroidetes	p	0.6228	0.2080	0.2657	0.5441	0.7040	0.2657	0.1108	0.1108	0.4685	0.2657	0.0724
	n	6	6	6	6	6	6	6	6	6	6	6
Firmicutes	r	-0.25714	0.60000	0.54286	0.31429	0.20000	0.54286	-0.71429	-0.71429	-0.37143	-0.54286	-0.77143
Firmicutes	p	0.6228	0.2080	0.2657	0.5441	0.7040	0.2657	0.1108	0.1108	0.4685	0.2657	0.0724
	n	6	6	6	6	6	6	6	6	6	6	6
Verrucomicrobia	r	-0.77143	0.77143	0.60000	0.60000	0.71429	0.94286	-0.88571	-0.88571	-0.65714	-0.94286	-0.71429
Verrucomicrobia	p	0.0724	0.0724	0.2080	0.2080	0.1108	0.0048	0.0188	0.0188	0.1562	0.0048	0.1108
	n	6	6	6	6	6	6	6	6	6	6	6
F_B_ratio	r	-0.25714	0.60000	0.54286	0.31429	0.20000	0.54286	-0.71429	-0.71429	-0.37143	-0.54286	-0.77143
F-B ratio	p	0.6228	0.2080	0.2657	0.5441	0.7040	0.2657	0.1108	0.1108	0.4685	0.2657	0.0724
	n	6	6	6	6	6	6	6	6	6	6	6
Bacteroidales_f__S24_7	r	0.48571	-0.65714	-0.71429	-0.42857	-0.37143	-0.71429	0.88571	0.88571	0.60000	0.71429	0.82857
Bacteroidales;f__S24-7	p	0.3287	0.1562	0.1108	0.3965	0.4685	0.1108	0.0188	0.0188	0.2080	0.1108	0.0416
	n	6	6	6	6	6	6	6	6	6	6	6
Lachnospiraceae	r	-0.88571	0.54286	0.88571	0.71429	0.77143	0.71429	-0.77143	-0.77143	-0.94286	-0.71429	-0.60000
Lachnospiraceae	p	0.0188	0.2657	0.0188	0.1108	0.0724	0.1108	0.0724	0.0724	0.0048	0.1108	0.2080
	n	6	6	6	6	6	6	6	6	6	6	6
Clostridiales_f__	r	-0.37143	0.54286	0.65714	0.42857	0.31429	0.48571	-0.65714	-0.65714	-0.42857	-0.48571	-0.71429
Clostridiales;f__	p	0.4685	0.2657	0.1562	0.3965	0.5441	0.3287	0.1562	0.1562	0.3965	0.3287	0.1108
	n	6	6	6	6	6	6	6	6	6	6	6
Oscillospira	r	0.25714	0.25714	0.14286	-0.14286	-0.31429	0.14286	-0.37143	-0.37143	-0.08571	-0.14286	-0.48571
Oscillospira	p	0.6228	0.6228	0.7872	0.7872	0.5441	0.7872	0.4685	0.4685	0.8717	0.7872	0.3287
	n	6	6	6	6	6	6	6	6	6	6	6
Lachnospiraceae_g__	r	-0.77143	0.08571	0.54286	0.48571	0.65714	0.37143	-0.31429	-0.31429	-0.60000	-0.37143	-0.02857
Lachnospiraceae;g__	p	0.0724	0.8717	0.2657	0.3287	0.1562	0.4685	0.5441	0.5441	0.2080	0.4685	0.9572
	n	6	6	6	6	6	6	6	6	6	6	6
Akkermansia	r	-0.77143	0.77143	0.60000	0.60000	0.71429	0.94286	-0.88571	-0.88571	-0.65714	-0.94286	-0.71429
Akkermansia	p	0.0724	0.0724	0.2080	0.2080	0.1108	0.0048	0.0188	0.0188	0.1562	0.0048	0.1108
	n	6	6	6	6	6	6	6	6	6	6	6
